# Entropy Generation Due to the Heat Transfer for Evolving Spherical Objects

**DOI:** 10.3390/e20080562

**Published:** 2018-07-28

**Authors:** Ho-Young Kwak

**Affiliations:** 1 Mechanical Engineering Department, Chung-Ang University, Seoul 06974, Korea; kwakhy@cau.ac.kr; 2 Blue Economy Strategy Institute Co. Ltd., Focus Buld. 23-10 Hyoryeong-ro, 60-gil, Seocho-gu, Seoul 06721, Korea

**Keywords:** bubble, heat transfer, entropy generation, lost work, supernova

## Abstract

Heat transfer accompanying entropy generation for the evolving mini and microbubbles in solution is discussed based on the explicit solutions for the hydrodynamic equations related to the bubble motion. Even though the pressure difference between the gas inside the bubble and liquid outside the bubble is a major driving force for bubble evolution, the heat transfer by conduction at the bubble-liquid interface affects the delicate evolution of the bubble, especially for sonoluminescing the gas bubble in sulfuric acid solution. On the other hand, our explicit solutions for the continuity, Euler equation, and Newtonian gravitational equation reveal that supernovae evolve by the gravitational force radiating heat in space during the expanding or collapsing phase. In this article, how the entropy generation due to heat transfer affects the bubble motion delicately and how heat transfer is generated by gravitational energy and evolving speed for the supernovae will be discussed. The heat transfer experienced by the bubble and supernovae during their evolution produces a positive entropy generation rate.

## 1. Introduction

Heat transfer through a finite temperature difference, a typical irreversible process, accompanies entropy generation, which produces the lost work in ordinary thermal systems in general [1] and damped oscillation for bubbles particularly [2]. For a microbubble oscillating in liquid, its behavior may be affected delicately by the entropy generation due to the heat transfer through the system boundary. In fact, various nonlinear phenomena appear for sonoluminescing gas bubbles in sulfuric acid solutions [3]. The conventional approach of polytropic approximation for the bubble behavior fails to account for the nonlinear behavior due to entropy generation because P_b_dV is an exact differential and its integral over a cycle of oscillation vanishes [4].

On the other hand, the amount of heat transfer that occurred during the evolution of the Newtonian stars or supernovae may be calculated if one knows the evolution of the system. Heat transfer for the stars is dependent on its gravitation energy and evolving speed [5], which is realized by the absorption of neutrinos during the expansion and generation of neutrinos during the collapsing phase [6]. So far, most of the work on the evolution of stars has employed the polytrope assumption for the whole star [7], which indicates that the star undergoes adiabatic expansion and contraction.

In this article, the entropy generation of evolving spherical objects such as mini or microbubbles in solutions and supernovae by heat transfer will be discussed with the help of the explicit solutions for the hydrodynamic equations such as mass, momentum and energy equations, and Newtonian gravity equations. The spherical objects considered in this study are the objects that expand or collapse homologously [5]. Homologous collapse or expansion, which has confirmed by the laser-induced bubble by controlling the laser energy input [8] means that every mass point inside the bubble or star may be traced back to a single point, i.e., the center of bubble or star, and vice versa. The entropy generation rate is always positive for the bubble whose evolution is affected by the heat transfer at the bubble-liquid interface as well as for the supernovae in which the amount of heat transfer is determined by the gravitational energy and evolving speed.

## 2. Bubble Evolution in Hot Liquid Medium

### 2.1. Evaporation of Liquid Droplet at Its Superheat Limit

One may heat a liquid droplet in an immiscible host liquid to a temperature far above its boiling point without the occurrence of a phase transition. The maximum temperature limit at which the liquid droplet evaporates explosively is called the superheat limit of liquid [9]. It has been observed that, when the temperature of a liquid droplet in the immiscible medium reaches its superheat limit at 1 atm, the droplet vaporizes explosively without the expansion of its volume and then the fully evaporated droplet becomes a bubble by the subsequent expansion [10].

Consider a butane bubble formed from the fully evaporated droplet at its superheat limit of 105 °C in ethylene glycol. The bubble of radius *R*(*t*) oscillates in hot ethylene glycol at ambient temperature *T*_∞_ and pressure *P*_∞_. When the bubble expands, the temperature of the vapor inside the bubble decreases and while the bubble contracts the temperature of the vapor increases. The heat transfer occurs due to such a temperature difference between the vapor inside the bubble (*T_b_*) and the surrounding liquid (*T*_∞_) through the thermal boundary layer.

The evaporated state which retains the volume of the saturated liquid state, having the effective volume of a liquid molecule, *V_m_* is characterized by its very high pressure, *P_n_* [11]

(1) $$P_{n}=\frac{Z\varepsilon_{m}}{3}/V_{m}$$

where *Z* is the number of the nearest neighboring molecules, e.g., *Z* = 12 for FCC lattice structure. The value of *P_n_* is approximately 138 bar for butane at its superheat limit. In Equation (1), *ε_m_* is the energy required to separate a pair of molecules from the given liquid state to the critical state. This is given approximately by [12]

(2) $$\varepsilon_{m}=4\varepsilon_{o}\left[1-{\left(\frac{\rho_{c}}{\rho_{m}}\right)}^{2}\right]\left[{\left(\frac{d_{w}}{d_{m}}\right)}^{6}-{\left(\frac{d_{w}}{d_{m}}\right)}^{12}\right]$$

The number density of the liquid, N, provides the average distance between molecules *d_m_* in Equation (2) and the effective molecular volume *V_m_* in Equation (1). The relation is

(3) $$\frac{\pi }{6}d_{m}^{3}N=V_{m}N=0.7405$$

where 0.7405 is the packing fraction of the FCC lattice structure. Since the internal pressure of the fully evaporated droplet is very large, as confirmed by Equation (1), the droplet expands spontaneously. At the initial stage of this process, the bubble expands linearly with time. However, its linear growing fashion slows down near the point where the nonlinear growing starts.

At the starting point of nonlinear growing, the bubble wall velocity vanishes [10]. Thus, it may be reasonable to choose this time as the starting point for the nonlinear bubble evolution. The pressure wave signal from the bubble at this point is given by [13]

(4) $$P_{non}^{\prime }=\frac{R_{non}}{r_{d}}\left[P_{b}(R_{non})-P_{\infty }\right]$$

One can calculate the pressure inside the bubble with the known value of the far-field pressure signal, *P’_non_*, and the bubble radius *R_non_* at the starting point of the nonlinear bubble motion. The physical properties such as pressure and temperature at this point may be used as the initial conditions for studying the subsequent nonlinear bubble evolution. The temperature at the starting point of nonlinear growing may be taken as the superheat limit of the liquid.

### 2.2. Thermal Boundary Layer Adjacent to the Bubble Wall

Assuming that the bubble is a spherical shape and that the instantaneous temperature profile at the thermal boundary layer adjacent to the bubble wall is quadratic [14], then we have

(5a) $$\frac{T-T_{\infty }}{T_{bl}-T_{\infty }}={\left(1-\xi \right)}^{2}$$

(5b) $$\xi =\frac{r-R_{b}}{\delta }$$

One can calculate the heat transfer rate if the boundary layer thickness, δ, is determined. The second-order curve for the temperature profile given in Equation (5a) satisfies the following boundary conditions that we might consider.

(6) $$T(R_{b},t)=T_{bl}\;\;\;\;T(R_{b}+\delta ,t)=T_{\infty }$$

and

(7) $${\left(\frac{\partial T}{\partial r}\right)}_{r=R_{b}+\delta }=0$$

**Figure 1:**
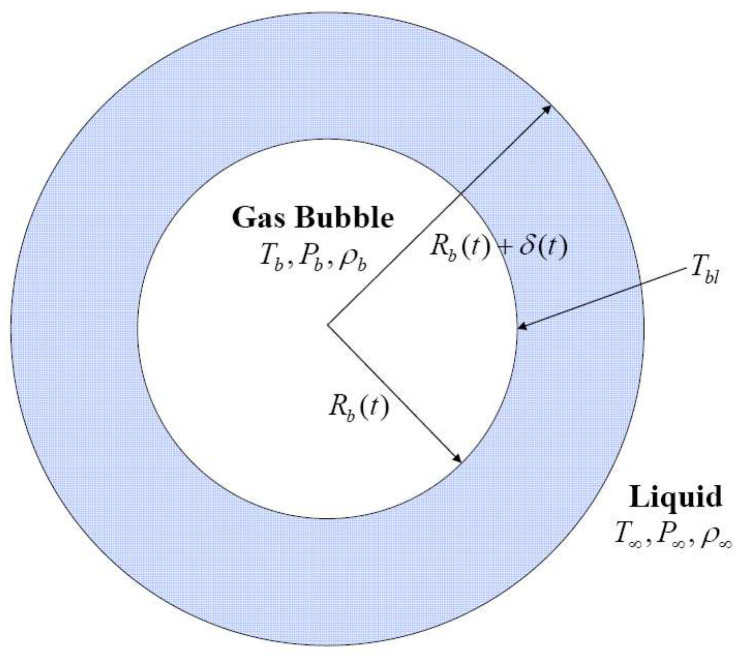
A model for a spherical bubble oscillating in a liquid.

A schematic of a bubble model is given in Figure 1, which shows a spherical gas or vapor bubble in liquid at an ambient temperature *T*_∞_ and pressure *P*_∞_. Heat transfer is assumed to occur through the thermal boundary layer of thickness *δ*(*t*). The driving force for the bubble evolution is either an initially imposed high pressure inside the bubble or an external force applied to the bubble. Assume that the spherical shape is maintained during bubble evolution.

### 2.3. Overall Energy Equation for a Bubble

If the bubble retains a thermal equilibrium for the gas inside the bubble, one may consider the following overall energy conservation for the bubble [15].

(8) dEdt+PbdVdt−dQdt=0

The first law of thermodynamics for the bubble, described in Equation (8), indicates that the sign convention of “heat transfer to the system” is positive. The ‘bar’ notation used in the heat transfer term indicates the physical quantity of the non-thermodynamic property. Assuming that the vapor inside the bubble obeys the ideal gas law, the internal energy of the vapor inside the bubble can be expressed as

(9) $$E=m_{b}C_{v,b}T_{b}=\frac{P_{b}V}{\gamma -1}$$

This equation implies that the gas is calorically perfect at equilibrium [16] as is assumed or the instantaneous temperature of the gas inside the bubble is spatially uniform, in which the equation of state for an ideal gas, *P_b_* = *ρ_g_R_g_T_b_* is valid. The heat transfer conducted through the thermal boundary layer can be obtained by applying the Fourier law at the bubble wall, or

(10) $$\dot{Q}=k_{l}4\pi R_{b}^{2}{\left(\frac{\partial T}{\partial r}\right)}_{r=R_{b}}=-\frac{8\pi R_{b}^{2}(T_{b}-T_{\infty })}{\delta }$$

Substituting Equations (9) and (10) into Equation (8), we have the time-dependent pressure inside the bubble, given as

(11) $$\frac{dP_{b}}{dt}=-\frac{3\gamma P_{b}}{R_{b}}\frac{dR_{b}}{dt}-\frac{6(\gamma -1)k_{l}(T_{b}-T_{\infty })}{\delta R_{b}}$$

The time dependent temperature of the vapor inside the bubble can be obtained from Equation (8) and the ideal gas law.

(12) $$\frac{dT_{b}}{dt}=-\frac{3(\gamma -1)T_{b}}{R_{b}}\frac{dR_{b}}{dt}-\frac{6(\gamma -1)k_{l}T_{b}(T_{b}-T_{\infty })}{\delta R_{b}P_{b}}$$

Equations (11) and (12) tell us that the adiabatic behavior is valid without the heat transfer term.

### 2.4. Bubble Wall Motion

The liquid may be considered incompressible because the wall velocity of the bubble formed from the evaporated droplet is much smaller than the sound speed of the liquid. With this assumption, one may obtain the well-known Rayleigh equation from the mass and momentum equations for the liquid. The following Rayleigh equation provides the radial motion of a spherical bubble in an unbounded liquid [17].

(13) $$R_{b}\frac{dU_{b}}{dt}+\frac{3}{2}U_{b}^{2}=\frac{1}{\rho_{\infty }}(P_{b}-P_{\infty })$$

The effects of surface tension and viscosity on the momentum equation may be neglected because the pressure terms due to the surface tension and viscosity in Equation (13) are negligible compared to the internal pressure of the bubble, *P_b_*, for the millimeter-sized bubble. The bubble wall velocity *U_b_* is the time derivative of the bubble radius:

(14) $$\frac{dR_{b}}{dt}=U_{b}$$

### 2.5. Energy Equation for the Liquid Adjacent to the Bubble Wall: The Thermal Boundary Layer

One may solve the following energy equation for the liquid adjacent to the bubble wall to obtain the thermal boundary layer thickness.

(15) $$\frac{dT}{dt}+u_{r}\frac{\partial T}{\partial r}=\frac{\alpha_{l}}{r^{3}}\frac{\partial }{\partial r}\left(r^{2}\frac{\partial T}{\partial r}\right)$$

where *α_l_* = *k_l_*/(*ρ*_∞_*C_l_*) is the thermal diffusivity. The radial velocity of liquid due to the bubble motion can be obtained from mass conservation for an incompressible liquid. That is

(16) $$u_{r}={\left(\frac{R_{b}}{r}\right)}^{2}U_{b}$$

Integrating Equation (15) from *r* = *R_b_* to *r* = *R_b_* + *δ* yields [13]

(17) $$\left[1+\frac{\delta }{R_{b}}+\frac{3}{10}{\left(\frac{\delta }{R_{b}}\right)}^{2}\right]\frac{d\delta }{dt}=\frac{6\alpha_{l}}{\delta }-\left[\frac{2\delta }{R_{b}}+\frac{1}{2}{\left(\frac{\delta }{R_{b}}\right)}^{2}\right]\frac{dR_{b}}{dt}-\delta \left[1+\frac{\delta }{2R_{b}}+\frac{1}{10}{\left(\frac{\delta }{R_{b}}\right)}^{2}\right]\frac{1}{T_{b}-T_{\infty }}\frac{dT_{b}}{dt}$$

Equation (17) provides the time rate change of the thermal boundary layer thickness, from which one can calculate the instantaneous heat transfer rate using Equation (10). One can solve Equations (11)–(14) and (17) simultaneously to obtain the next time step value of *P_b_*, *T_b_*, *U_b_*, *R_b_* and *δ* by the Runge-Kutta numerical method with appropriate initial conditions.

### 2.6. Entropy Generation during Bubble Evolution

For the evolving bubble formed from a fully evaporated liquid droplet at its superheat limit, the heat transfer mechanism is clearly defined. Using the thermal boundary layer with constant thickness at the bubble-liquid interface, the thermal damping due to heat exchange with the surrounding liquid was first treated by Moody [2]. The damped bubble oscillation due to heat transfer through the bubble wall simply displays an available power loss due to entropy production. The entropy generation rate in such an oscillating bubble-liquid system is the combination of the rate change of entropy for vapor inside the bubble due to bubble pulsation and the net entropy flow out of the bubble as the result of the heat exchange [13]. That is,

(18) S˙gen=DSbDt−1T∞dQdt=dQdt(−1Tb+1T∞)∝(Tb−T∞)2

Equation (18) shows that the entropy generation due to the transfer at the bubble wall during the bubble evolution is always positive. The lost work, which is proportional to the entropy generation (Guoy-Stodolar theorem, [1]), induces thermal damping in the bubble motion.

### 2.7. Evolution of a Bubble Formed from a Droplet at Its Superheat Limit in Hot Liquid Medium

**Figure 2:**
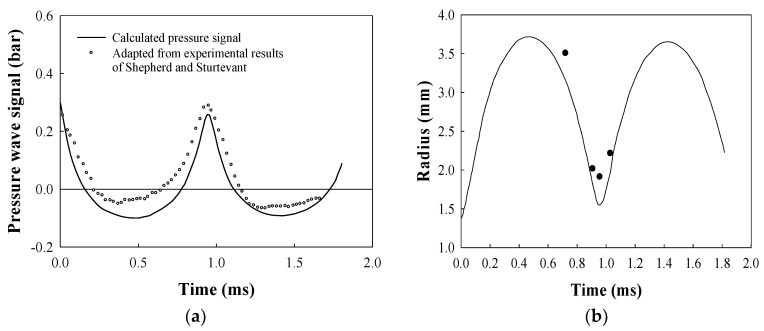
(**a**) The far-field pressure signal from the oscillating butane bubble in ethylene glycol; (**b**) The radius–time curve for a bubble evolved from a butane droplet at its superheat limit in ethylene glycol.

**Figure 3:**
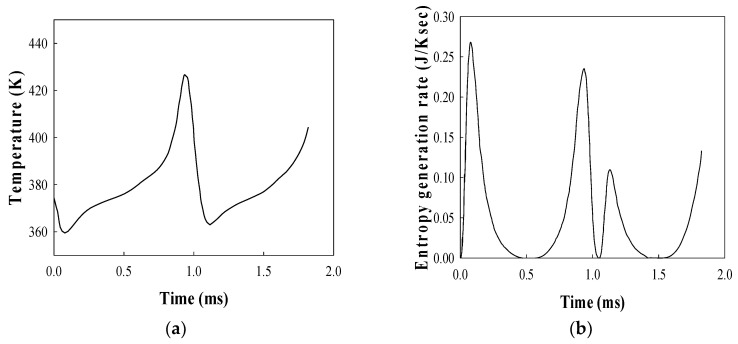
(**a**) The time rate change of the temperature for the vapor inside the butane bubble; (**b**) The entropy generation rate for the butane bubble shown in Figure 2.

The calculated far-field pressure wave signals from the evolving butane bubble in ethylene glycol at the ambient pressure of 1 atm and at a liquid temperature of 378 K are shown in Figure 2a. The initial conditions for nonlinear bubble evolution are chosen to be *R_o_* = 1.37 mm, *P_b_* (*R_o_*) = 6.67 bar and *T_b_* = 378 K, the superheat limit of butane. The empty circles in this figure denote the experimental data by Shepherd and Sturtevant [10]. Figure 2b displays the instantaneous bubble radius during the evolution of a butane bubble. Full circles indicate some of the observed data [10]. The time rate change of the vapor temperature inside the bubble is shown in Figure 3a, which shows that the bubble evolution is neither isothermal nor adiabatic. The conventional method of polytropic assumption, which produces an isothermal bubble or an adiabatic bubble, cannot catch this bubble behavior. In Figure 3b, the entropy generation rate experienced by the butane bubble is shown. As expected, the entropy generation during the bubble oscillation is always positive. Thermal damping due to finite heat transfer [13] is barely seen in the bubble’s radius–time curve shown in Figure 2b.

Bubble dynamics formulated in this section contain the thermal behavior for the vapor inside the bubble in oscillation for a particular case of ‘uniform temperature limit’, which is appropriate for the case in which the characteristic time of the bubble evolution (ms) is much longer than the relaxation time of the translational motion of the vapor molecules [16].

## 3. Microbubble Behavior under an Ultrasonic Field

### 3.1. Mass, Momentum and Energy Conservations for Gas inside the Bubble

A microbubble trapped under an ultrasonic field oscillates, synchronizing with the applied ultrasound whose frequency is around 25 kHz [18]. With appropriate conditions such as a suitable bubble radius and the driving amplitude of the ultrasound, the microbubble emits a light at the collapse, which is called “sonoluminescence”. The characteristic time of the microbubble evolution under ultrasound is a microsecond so that the temperature distribution inside the bubble may not be uniform near the bubble collapse. One may solve the Navier-Stokes equations for the gas inside the bubble and the liquid adjacent to the bubble wall to understand the microbubble behavior under ultrasound. The mass and momentum equations for the gas inside the bubble with spherical symmetry are given as

(19) $$\frac{\partial \rho_{g}}{\partial t}+\frac{1}{r^{2}}\frac{\partial }{\partial r}\left(\rho_{g}u_{g}r^{2}\right)=0,$$

(20) $$\frac{\partial }{\partial t}\left(\rho_{g}u_{g}\right)+\frac{1}{r^{2}}\frac{\partial }{\partial r}\left(\rho_{g}u_{g}^{2}r^{2}\right)+\frac{\partial P_{b}}{\partial r}=0,$$

A set of analytical solutions for the above conservation equations [19] is given as

(21) $$\rho_{g}=\rho_{o}+\rho_{r}$$

(22) $$u_{g}=\frac{{\dot{R}}_{b}}{R_{b}}r,$$

(23) $$P_{b}=P_{bo}-\frac{1}{2}\left(\rho_{o}+\frac{1}{2}\rho_{r}\right)\frac{{\ddot{R}}_{b}}{R_{b}}r^{2},$$

where *ρ_o_*(*R_o_*)^3^ = constant and *ρ_r_* = *ar*^2^/(*R_b_*)^5^. The constant a is related to the gas mass inside a bubble by *a*/*m* = 5(1 − *N_BC_*)/(4*π*) with *N_BC_* = [*P_bo_*(*R_b_*)^3^/*T_bo_*]/[*P*_∞_(*R_o_*)^3^/*T*_∞_]. The subscript, o, denotes the properties at the bubble center. The linear velocity profile describes the homologous motion of a spherical object, which was verified experimentally by the laser-induced bubble in water [8]. The linear velocity profile obtained from the continuity equation, which makes the viscosity term vanish in the momentum equation, implies that the bubble collapses or expands homologously [5]. The quadratic pressure profile given in Equation (23), was verified by comparisons with direct numerical simulations [20].

Assuming that the internal energy for the gas inside a bubble is a function of gas temperature only as *de* = *C_v_*,*_b_dT_b_*, the energy equation for the gas inside the bubble may be written as

(24a) $$\rho_{g}C_{v,b}\frac{DT_{b}}{Dt}=-\frac{P_{b}}{r^{2}}\frac{d}{dr}\left(r^{2}u_{g}\right)-\frac{1}{r^{2}}\frac{d}{dr}\left(r^{2}q_{r}\right),$$

The viscous dissipation term in the internal energy equation also vanishes because the radial component of the stress is null with the linear velocity profile. Since the solutions for the mass and momentum equations, which are given in Equations (21)–(23), also satisfy the kinetic energy equation; only the internal energy equation given in Equation (24a) needs to be solved. Using the definition of enthalpy, the internal energy equation for the gas can be also written as

(24b) $$\rho_{g}C_{P,b}\frac{DT_{b}}{Dt}=\frac{DP_{b}}{Dt}-\frac{1}{r^{2}}\frac{\partial }{\partial r}\left(r^{2}q_{r}\right).$$

Eliminating *DT_b_*/*Dt* from Equation (24a,b), one can obtain the following heat transport equation for the gas inside bubble [19].

(25) $$\frac{DP_{b}}{Dt}=-\frac{\gamma P_{b}}{r^{2}}\frac{\partial }{\partial r}\left(r^{2}u_{g}\right)-\frac{\left(\gamma -1\right)}{r^{2}}\frac{\partial }{\partial r}\left(r^{2}q_{r}\right).$$

With uniform pressure approximation, which is legitimate when the bubble wall acceleration is considerably less than 10^12^ m/s^2^ [21], Equation (25) can be written as follows with help of Equations (21)–(23)

(26) $$\frac{\left(\gamma -1\right)}{r^{2}}\frac{d}{dr}\left(r^{2}q_{o}\right)=-\left[\frac{dP_{bo}}{dt}+3\gamma P_{bo}\frac{{\dot{R}}_{b}}{R_{b}}\right]$$

A temperature profile can be obtained by solving Equation (26) with the Fourier law. That is [18],

(27) $$T_{b}(r)=\frac{B}{A}\cdot \left[-1+\sqrt{{\left(1+\frac{A}{B}T_{bo}\right)}^{2}-2\eta \frac{A}{B}(T_{bl}-T_{\infty }){\left(\frac{r}{R_{b}}\right)}^{2}}\right],$$

where *A* and *B* are the coefficients in the temperature-dependent gas conductivity, having a form such as *k_g_* = *AT* + *B* and *η* = (*R_b_*/*δ*)(*k_l_*/*B*). For air *A* = 5.528 × 10^−5^ J/msK^2^ and *B* = 1.165 × 10^−2^ J/msK, and for argon A = 2.65 × 10^−5^ J/msK^2^ and B = 1.347 × 10^−3^ J/msK were used [22]. The temperature distribution given in Equation (27) is valid until the characteristic time of the bubble evolution is an order of the relaxation time for the vibrational motion of the molecules [16] and/or is much less than the relaxation time of the translational motion of the molecules [23]. The temperature at the bubble wall *T_bl_* can be obtained from Equation (27). Such a uniform pressure approximation inside the bubble can appropriately describe the sonoluminescing gas bubble in sulfuric acid solutions [24].

### 3.2. Bubble Wall Motion under an Ultrasonic Field

The bubble speed at the bubble collapse for a sonoluminescing gas bubble in water is over 600 m/s [25] so that one considers the compressible effect for the bubble motion. The mass and momentum equation outside the bubble wall, accounting for the compressible effect of the liquid, provides the well-known Keller-Miksis equation for the bubble wall motion [26], which is valid until the bubble wall velocity is less than the sound speed of the liquid. That is

(28) $$R_{b}\left(1-\frac{U_{b}}{C_{B}}\right)\frac{dU_{b}}{dt}+\frac{3}{2}U_{b}^{2}\left(1-\frac{U_{b}}{3C_{B}}\right)=\frac{1}{\rho_{\infty }}\left(1+\frac{U_{b}}{C_{B}}+\frac{R_{b}}{C_{B}}\frac{d}{dt}\right)\left[P_{B}-P_{s}\left(t+\frac{R_{b}}{C_{B}}\right)-P_{\infty }\right]$$

The liquid pressure on the external side of the bubble wall *P_B_* is related to the gas or vapor pressure inside the bubble wall *P_b_* by

(29) $$P_{B}=P_{b}-\frac{2\sigma }{R_{b}}-4\mu \frac{U_{b}}{R_{b}}$$

The pressure of the deriving sound field *P_s_* may be represented by a sinusoidal function such as *P_s_* = −*P_A_*sin*ωt* where *ω* = 2*πf_d_* and *f_d_* is the driving frequency.

The heat transfer occurs inside the bubble as well as at the thermal boundary layer adjacent to the bubble wall for cases in which the gas or vapor temperature inside the bubble is not uniform. The equations for the time rate change of the pressure, the temperature for the vapor inside the bubble, and the time rate change of the thermal boundary layer equation for a perfect equilibrium bubble, which are given in Equations (11), (12), and (17), respectively, should be replaced by the following equations.

(30) $$\frac{dP_{b}}{dt}=-\frac{3\gamma P_{b}}{R_{b}}\frac{dR_{b}}{dt}-\frac{6(\gamma -1)k_{l}(T_{bl}-T_{\infty })}{\delta R_{b}}$$

(31) $$\frac{dT_{bo}}{dt}=-\frac{3(\gamma -1)T_{bo}}{R_{b}}\frac{dR_{b}}{dt}-\frac{6(\gamma -1)k_{l}T_{bo}(T_{bl}-T_{\infty })}{\delta R_{b}P_{b}}$$

(32) $$\left[1+\frac{\delta }{R_{b}}+\frac{3}{10}{\left(\frac{\delta }{R_{b}}\right)}^{2}\right]\frac{d\delta }{dt}=\frac{6\alpha_{l}}{\delta }-\left[\frac{2\delta }{R_{b}}+\frac{1}{2}{\left(\frac{\delta }{R_{b}}\right)}^{2}\right]\frac{dR_{b}}{dt}-\delta \left[1+\frac{\delta }{2R_{b}}+\frac{1}{10}{\left(\frac{\delta }{R_{b}}\right)}^{2}\right]\frac{1}{T_{bl}-T_{\infty }}\frac{dT_{bl}}{dt}$$

One can solve Equations (14), (28), and (30)–(32) simultaneously to obtain the next time step value of *R_b_*, *U_b_*, *P_b_*, *T_o_* and *δ*, respectively. The instantaneous temperature distribution can be obtained from Equation (27).

### 3.3. Sonoluminescing Bubble in Sulfuric Acid Solutions

**Figure 4:**
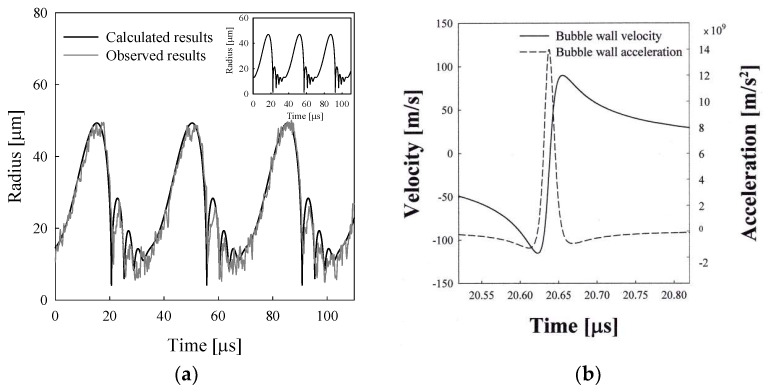
(**a**) The theoretical radius–time curve and (**b**) bubble wall velocity and acceleration around the collapse point for an argon bubble of *R_o_* = 13.0 μm at *P_A_* = 1.40 atm and *f_d_* = 28.5 kHz in a sulfuric acid solution. The inset in Figure 4a shows the bubble radius–time curve with a polytropic relationship.

The bubble behavior model discussed in Section 3.1 and Section 3.2 adequately describes a sonoluminescing bubble in sulfuric acid solutions, which reveals various nonlinear phenomena. The calculated radius–time curve for an argon bubble with an equilibrium radius *R_o_* of 13 μm driven by the ultrasound with a frequency of 28.5 kHz and amplitude of 1.4 atm in an aqueous solution of sulfuric acid is shown in Figure 4a, which shows close agreement between the calculation results and observed ones. The thermodynamic properties for an 85% sulfuric acid solution are *ρ* = 1800 kg/m^3^, *C_s_* = 1470 m/s, *μ* = 0.025 Ns/m^2^, *σ* = 0.055 N/m, *k_l_* = 0.4 W/mK, and *C_p,l_* = 1817 J/kgK. The sound speed in an 85% sulfuric acid solution is comparable to the sound speed in water. In fact, the sound speed is one of the decisive properties for the occurrence of the sonoluminescence [27].

The calculated bubble radius–time curve along with the observed data which were obtained originally by Flannigan et al. [28] is plotted in Figure 4a using a free data digitizer (WinDIG 2.5). The bubble emits a nanosecond flash every cycle at the collapse point [29]. The bubble with an equilibrium radius of 5–7 μm emits picosecond flash every cycle in water [30] as well as in sulfuric acid solution [29]. The calculation result with the polytropic relation shows a smaller bouncing motion in magnitude as shown in the inset of Figure 4a. Figure 4b shows the instantaneous bubble wall velocity and the bubble wall acceleration around the collapse point for the bubble shown in Figure 4a. The absolute value of the calculated minimum velocity at the collapse point is about 120 m/s, which is close to the observed value of 100 m/s. On the other hand, the absolute value of the minimum velocity obtained by using the polytropic relationship is about 460 m/s, which is much higher than the observed value. Consequently, the calculation results by polytropic relation provide a considerably smaller minimum bubble radius at the collapse point. As shown in Figure 4b, the calculated maximum bubble wall acceleration in a sulfuric acid solution is about 1.2 × 10^10^ m/s^2^, which is much smaller than the case of the sonoluminescing gas bubble in water, by two orders of magnitude.

**Figure 5:**
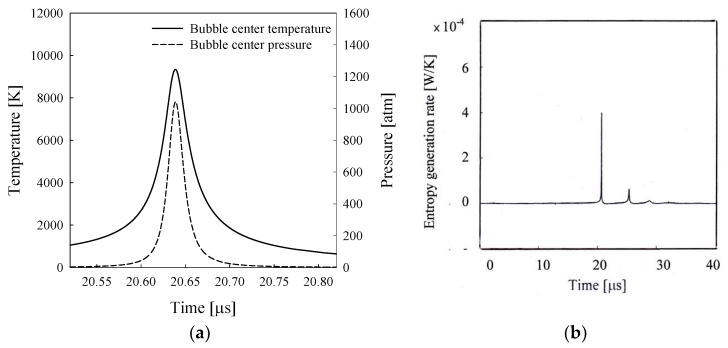
(**a**) The time-dependent gas temperature and pressure at the bubble center during the collapse phase and (**b**) the entropy generation rate for the bubble shown in Figure 4a.

Figure 5a shows the calculated time-dependent temperatures and pressures at the center of the bubble shown in Figure 4a. The calculated peak temperature at the collapse point, which is about 9300 K, is close to the observed value of 10,000 K [28]. Additionally, the calculated peak pressure value of 1020 atm is close to the observed value of 1090 atm. The estimated peak temperature and pressure at the collapse point are approximately 25,000 K and 10,000 atm, respectively, for the sonoluminescing air bubble in water [21]. The approximation of ‘uniform pressure’ turns out to be good for the sonoluminescing bubble in sulfuric acid solution because the bubble wall acceleration at the collapse point is much smaller than that of the sonoluminescing gas bubble in water. Figure 5b shows the entropy generation rate, which shows a sharp peak at the first collapse and a subsequent smaller peak during the rebounding motion. This figure indicates that heat transfer occurs notably around the first collapse phase.

**Figure 6:**
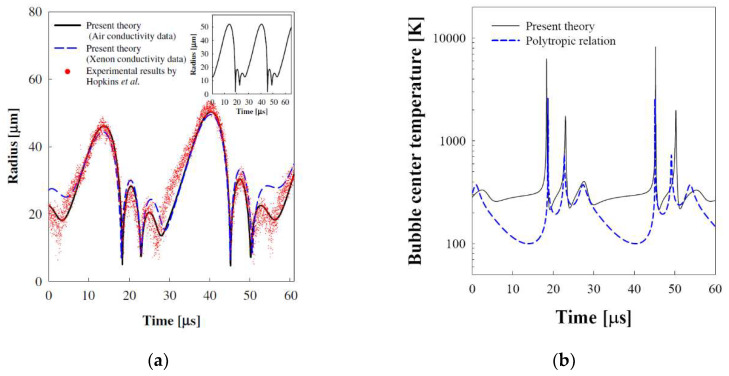
(**a**) The theoretical radius–time curve along with observed values by Hopkins et al. [3] and (**b**) the time-dependent center temperature for the xenon bubble of *R_o_* = 15.0 μm at *P_A_* = 1.50 atm and *f_d_* = 37.8 kHz in a sulfuric acid solution.

Slight changes in the equilibrium radius of the bubble and driving amplitude and the frequency of the applied ultrasound induce a drastic change for the bubble motion in a sulfuric acid solution. The calculated radius–time curve along with the observed results for a xenon bubble with *R_o_* = 15 mm, driven by the ultrasonic field with a frequency 37.8 kHz and amplitude of 1.5 atm in an 85% sulfuric acid solution is shown in Figure 6a. With air thermal conductivity (solid line), the calculated radius–time curve which exactly mimics the alternating pattern of the observed result shows two states of bubble motion, a light emission cycle after no light emission cycle. With xenon thermal conductivity (dashed line), however, a slightly different pattern for the bubble motion was obtained. The time-dependent temperature at the bubble center, as shown in Figure 6b, indicates also the two states of bubble motion. The Rayleigh–Plesset equation with polytropic relation, a conventional method used to predict the sonoluminescence phenomena [31,32], cannot predict the two states of bubble motion as shown in the inset in Figure 6a.

**Figure 7:**
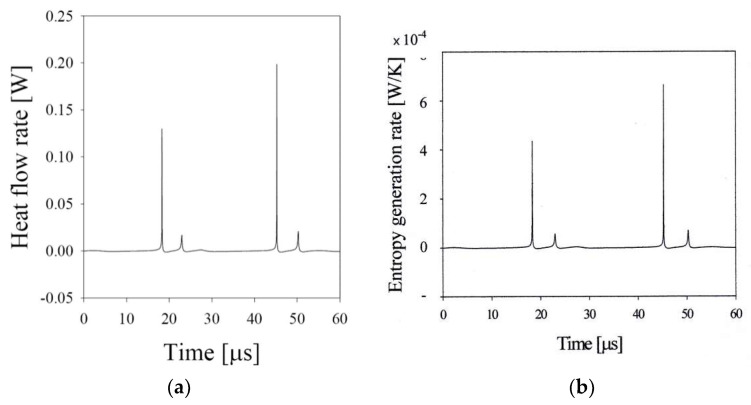
(**a**) The heat flow rate and (**b**) the corresponding entropy generation rate for the xenon bubble of *R_o_* = 15.0 μm at *P_A_* = 1.50 atm and *f_d_* = 37.8 kHz in a sulfuric acid solution.

These calculated results imply that the bubble behavior an consequently the sonoluminescence phenomena depends crucially on the heat transfer experienced by the bubble as shown in Figure 7a. At the sonoluminescing phase, the heat flow rate is 0.20 W at the collapse point. On the other hand, the heat flow rate is approximately 0.14 W at the collapse point for the non-lighting phase. The entropy generation rate is similar to the pattern of the heat flow rate as shown in Figure 7b. The alternating pattern for the bubble motion may happen due to the entropy generation by the heat transfer through the bubble wall [24], which produces lost work: less entropy generation in one cycle having a lower maximum bubble radius and, consequent, a lower minimum bubble radius at the collapse provides more expansion work for the bubble’s next cycle, while a larger amplitude motion experiencing more entropy generation provides less expansion work to the subsequent motion. The calculated minimum bubble radius for the light-emitting cycles, 4.6 μm, is close to the observed value of 3.7 μm [3].

**Figure 8:**
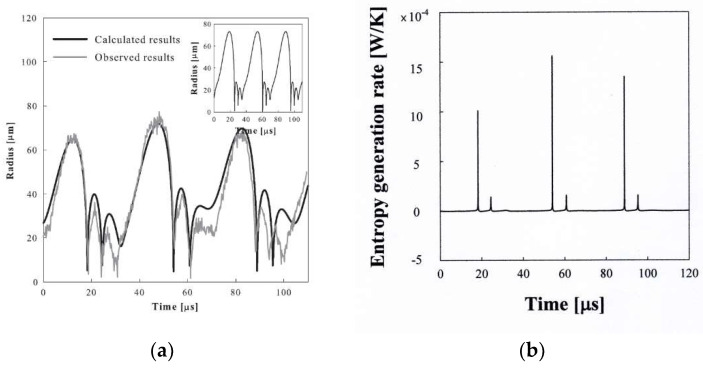
(**a**) A theoretical radius–time curve for an argon bubble of *R_o_* = 17.0 μm at *P_A_* = 1.72 bar and *f_d_* = 28.5 kHz in a sulfuric acid solution. The insert shows the bubble radius–time curve with a polytropic relationship; (**b**) The rntropy generation rate for the bubble shown in Figure 8a.

Figure 8a shows the bubble radius–time curve for an argon bubble of *R_o_* = 17μm driven with an ultrasound amplitude of 1.7 atm and a frequency of 28.5 kHz [33], which mimics the observed one remarkably [3]. This is a case in which the bubble emits light for two consecutive cycles after a no-emission cycle. The calculation result with a polytropic relation shows the same pattern of bubble behavior every cycle as shown in the inset of Figure 8a. The entropy generation rate due to the heat transfer through the wall for the bubble shown in Figure 8a, which can be obtained from Equation (18), is given in Figure 8b. Less entropy generation in one cycle provides more expansion work for the bubble’s next cycle. Such an alternative motion is possible because the same ultrasound amplitude is applied each cycle.

## 4. Evolution of Stars

Cosmological fluid equations (continuity, Euler, and Newtonian gravity) were first proposed by Sir James Jeans [34] as a theory of galaxy formation. The stellar stability, spherical oscillation, and gravitational collapse of Newtonian stars such as the Sun, Jupiter, and Saturn could be obtained by solving the cosmological fluid equations [5]. It is very interesting to consider the heat transfer mechanism occurred during the evolution of such astronomical objects, which have been done numerically by many researchers. For examples, the radiation hydrodynamics code coupled with neutron transport [35] and a sophisticated two-dimensional, time-dependent, multi-group, and multi-angle radiation hydrodynamics numerical scheme [36] were developed for the core-collapse supernova simulation. Buras et al. [37] used mass, momentum and energy equation in spherical coordinates and azimuthal symmetry in their hydrodynamic simulations for the core-collapse supernovae. The Newtonian gravity term and the neutrino interaction term for momentum transfer were included in the momentum equation. Instead of the heat flow term in the energy equation, neutron source terms for energy exchange were included. Murphy and Burrow [38] used continuity, Euler, and energy equations in their simulation. The Newtonian gravity term was included in the momentum equation. However, the term representing heat flow in the energy equation was replaced as the neutrino heating and cooling terms which are dependent on the electron neutrino temperature and the neutron and proton fractions. However, the core-collapse supernova mechanism has never been studied by hydrodynamic equations with keeping the heat flow term in the energy equation, which may provide a simple and quantitative theory of the supernova explosion.

### 4.1. Hydrodynamics for the Stars

The equation of continuity, the Euler equation for an irrotational fluid, and Newtonian gravity are given below.

(33) $$\frac{\partial \rho }{\partial t}+\nabla \cdot (\rho \vec{u})=0$$

(34) $$\frac{\partial \vec{u}}{\partial t}+\vec{u}\cdot \nabla \vec{u}=-\frac{1}{\rho }\nabla P-\nabla \phi$$

(35) $$\nabla^{2}\phi =4\pi G\rho$$

In these equations, *ρ* is the density, *P* is the pressure, u is the fluid velocity, *ϕ*(*r,t*) is gravitational potential, and *G* is the gravitational constant. 

From the continuity equation, the following linear velocity and quadratic density profiles can be obtained as a lowest-order approximation. The full derivations of these equations can be found in the literature [19,21].

(36) $$u=\dot{R}r/R$$

(37) $$\rho (r,t)=\frac{b}{R^{3}(t)}+\frac{ar^{2}}{R^{5}(t)}$$

where *R* is the radius of the star, *dR*/*dt* is the velocity of the star, and “*a*” and “*b*” are constants which will be identified later.

With the density profile given in Equation (37) and the velocity *u* in Equation (36), the gravitational potential *ϕ*(*r,t*) and the pressure *P*(*r,t*) can be obtained by solving the Poisson and Euler equations. These are in terms of a and b, given as follows.

(38) $$\phi (r,t)=\phi_{o}(t)+\left(\frac{2\pi Gb}{3}\frac{r^{2}}{R^{3}}+\frac{\pi Ga}{5}\frac{r^{4}}{R^{5}}\right)$$

(39) $$P(r,t)=P_{o}(t)-\left[\left(\frac{b}{2}\frac{r^{2}}{R^{2}}+\frac{a}{4}\frac{r^{4}}{R^{4}}\right)\frac{\ddot{R}}{R^{2}}+\left(\frac{2\pi Gb^{2}}{3}\frac{r^{2}}{R^{2}}+\frac{8\pi Gab}{15}\frac{r^{4}}{R^{4}}+\frac{2\pi Ga^{2}}{15}\frac{r^{6}}{R^{6}}\right)\frac{1}{R^{4}}\right]$$

At the center of the star, the above equation can be arranged as follows after applying the boundary conditions, *P* = 0 and *ϕ* = −*GM*/*R* at *r* = *R*.

(40) $$\phi_{o}=-\frac{GM}{R}-\frac{\pi G(3a+10b)}{15}\frac{1}{R}$$

and

(41) $$P_{o}(t)=\left(\frac{a+2b}{4}\right)\frac{\ddot{R}}{R^{2}}+\frac{2\pi G(a^{2}+4ab+5b^{2})}{15}\frac{1}{R^{4}}$$

To determine the values of “*a*” and “*b*” in Equation (37), an equation of state for the gas inside the star is required. Excluding the viscous dissipation term, the energy equation with internal energy and enthalpy representations can be written as follows [13,19]

(42) $$\rho C_{v}\frac{DT}{Dt}=-P\nabla \cdot \vec{u}-\nabla \cdot \vec{q}$$

(43) $$\rho C_{p}\frac{DT}{Dt}=\frac{DP}{Dt}-\nabla \cdot \vec{q}$$

In the above equations, *T* is the temperature, and *C_v_* and *C_p_* are the specific heats at a constant volume and constant pressure, respectively, and *q* is the heat flux.

From Equations (42) and (43), one can obtain the following equation by eliminating the term *DT*/*Dt*.

(44) $$(\gamma -1)\nabla \cdot \vec{q}=-\frac{DP}{Dt}-\frac{3\gamma P\dot{R}}{R}$$

where *γ* is the specific heat ratio.

The above equation indicates that the heat transport inside the star can be obtained if one knows the evolution of the star without the detailed mechanism of heat transfer, which can be possible only for an “ideal gas system”. On the other hand, by eliminating the term, $\nabla \cdot \vec{q}$, in Equations (42) and (43), one can obtain the temperature at the center of the star in terms of the pressure at the center and the radius.

(45) $$bR_{g}T_{o}(t)=P_{o}(t)R^{3}(t)$$

where *R_g_* is the gas constant and the subscript “*o*” denotes the property at the center.

Assuming that the “polytrope” only holds true at the center, the following equation can be applied,

(46) $$P_{0}=\kappa \rho_{0}^{\gamma }$$

The polytrope assumption at the center is equivalent to the assumption that the center is neither a heat source nor a heat sink, i.e., $\nabla \cdot \vec{q}=0$ at the center. This assumption, of course, is not valid when a nuclear reaction occurs inside the star.

Using the velocity, density, and pressure profiles given in Equations (36), (37) and (39), respectively, one can solve the energy equation given in Equation (42) or Equation (43) with the boundary condition given in Equation (46). These are

(47) a=−b=−15M/8

(48) $$T(r,t)=T_{o}-\left(\frac{R\ddot{R}}{4R_{g}}+\frac{2\pi Gb}{5R_{g}R}\right){\left(\frac{r}{R}\right)}^{2}+\frac{2\pi Gb}{15R_{g}R}{\left(\frac{r}{R}\right)}^{4}$$

where

(49) $$T_{o}=\frac{R\ddot{R}}{4R_{g}}+\frac{4\pi Gb}{15R_{g}R}$$

Using the polytrope assumption at the center and the explicit values of “*a*” and “*b*”, the profiles for pressure, temperature, density and gravitational potential in the star can be obtained [5]. These are

(50) $$P(r,t)=\frac{15M}{32\pi }\left(\frac{32\pi \kappa^{\prime }}{15M}\frac{1}{R^{3\gamma -4}}-GM{\left(\frac{r}{R}\right)}^{2}\right){\left(1-\frac{r^{2}}{R^{2}}\right)}^{2}\frac{1}{R^{4}}$$

(51) $$T(r,t)=\frac{1}{4R_{g}}\left(\frac{32\pi \kappa^{\prime }}{15M}\frac{1}{R^{3\gamma -4}}-GM{\left(\frac{r}{R}\right)}^{2}\right)\left(1-\frac{r^{2}}{R^{2}}\right)\frac{1}{R}$$

(52) $$\rho (r,t)=\frac{15M}{8\pi }\left(1-\frac{r^{2}}{R^{2}}\right)\frac{1}{R^{3}}$$

where $\kappa^{\prime }={\left(15M/8\pi \right)}^{\gamma }\kappa$.

It is noted that the above profiles for the pressure, temperature, and density satisfy the mass, momentum and energy equations and the ideal gas law. The requirement that the pressure and temperature should be greater than zero everywhere results in the following constraint for $\kappa^{\prime }$.

(53) $$\frac{32\pi \kappa \prime }{15M}\geq GMR^{3\gamma -4}$$

Such an ideal gas model of Newtonian stars provides the upper bound value of the Chandrasekhar mass for white dwarfs and the central densities, pressures, and temperatures of the stars such as Sun, Jupiter and Saturn [39].

### 4.2. Core-Collapse Implosion: Early Supernova

With the polytrope assumption at the center of the star, the equation for the pressure simplifies to the following equation of motion for the star.

(54) $$\frac{d^{2}}{dt^{2}}R(t)=\frac{32\pi \kappa^{\prime }}{15M}\frac{1}{R^{3\gamma -2}}-\frac{2GM}{R^{2}}$$

Thus, the continuity, Euler, Poisson, and energy equations with the polytrope assumption only at the center can be reduced to Equation (54), which can be solved using the standard “energy” method if Equation (54) is converted into the following form:

(55) $$\frac{1}{2}{\dot{R}}^{2}+W(R)=\varepsilon$$

where *ε* is a constant and the potential *V*(*R*) is given by Equation (56).

(56) $$W(R)=\frac{32\pi \kappa^{\prime }}{45M(\gamma -1)}\frac{1}{R^{3\gamma -3}}-\frac{2GM}{R}$$

The solution of Equation (54) and, hence, Equation (55) can then be given by the elementary integral shown in Equation (57).

(57) $$\int_{R_{\max}}^{R}\frac{dR}{\sqrt{2\left[\varepsilon -W(R)\right]}}=\pm t$$

where + and − correspond to the cases of expansion and contraction, respectively.

From Equation (56), it follows that there is a bound state for *γ* > 4/3 but there is no stable bound state for *γ* < 4/3. For the collapsing star, the potential should be less than zero so that the constant $\kappa^{\prime }$ satisfies the following constraint.

(58) $$GMR^{3\gamma -4}\leq \frac{32\pi \kappa \prime }{15M}\leq 6(\gamma -1)GMR^{3\gamma -4}$$

With the upper bound value of $\kappa^{\prime }$, *W*(*R*_max_) = 0, the equation of motion for the collapsing star, Equation (54), can be rewritten as

(59) $$\frac{d^{2}}{dt^{2}}R(t)=-(4-3\gamma )\frac{2GM}{R^{2}}$$

One can immediately obtain the following solutions for Equation (59).

(60) $$t=\frac{\psi +\sin\psi }{2k}$$

(61) $$R=\frac{1}{2}R_{\max}(1+\cos\psi )$$

where

(62) $$k=\sqrt{-2\varepsilon (R_{\max})}/R_{\max}$$

And

(63) $$\varepsilon (R_{\max})=-(4-3\gamma )2GM/R_{\max}$$

Note that the solutions given in Equations (60)–(63) coincide with those of the Oppenheimer–Snyder [40] equations of gravitational collapse. This coincidence stems from their oversimplified assumption of uniform density and zero pressure even though they started with general relativistic equations. Their general relativistic equation for the equation of motion for a star is given by

(64) $$\frac{d^{2}R}{dt^{2}}=-\frac{GM}{R^{2}}$$

Comparing Equation (64) with Equation (59), Equation (64) has no inner pressure force acting against the gravitational collapse so that the star governed by Equation (64) will collapse infinitely.

From Equations (42) and (43), their solutions, and Equations (50)–(52) for *P*, *T*, and *ρ*, respectively, the heat flow into a sphere of radius *r* < *R*(*t*) in a star during the expansion as well as contraction is given by [5]

(65) $$-\nabla \cdot \vec{q}=\frac{\left(4-3\gamma \right)}{4(\gamma -1)}\frac{GM^{2}}{R}\frac{15}{8\pi }{\left(1-\frac{r^{2}}{R^{2}}\right)}^{2}\left(\frac{r^{2}}{R^{2}}\right)\frac{\dot{R}}{R}\frac{1}{R^{3}}$$

Equation (65) indicates that the heat transfer rate is dependent on the velocity of the evolution and the gravitational energy. While a star is at a stable equilibrium, there is no heat flow in or out. For *γ* > 4/3, $-\nabla \cdot \vec{q}$ is negative when a star expands and positive when it contracts. Thus, during the expansion, the heat flows radially outwards in a star so that the pressure decreases, causing the gravitational attraction to dominate over the pressure, which prevents a star from expanding forever. On the other hand, during the contraction, the heat flows radially inwards so that the pressure increases dominantly over the gravitational attraction. This prevents a star from collapsing. The heat flow rate to the system with a sign convention of heat given in Equation (8) is given by

(66) dQdt=∫0R(−∇⋅q→)4πr2dr

For *γ* < 4/3, exactly the opposite takes place, i.e., $-\nabla \cdot \vec{q}$ is positive when a star expands, and is negative when it contracts. Thus, during expansion, the heat flows radially inwards so that the pressure increases in comparison with the gravitational attraction, which promotes the star‘s continued expansion. Conversely, when a star contracts, heat flows radially outwards so that the pressure decreases in comparison with the gravitational attraction, which furthers the star collapse. This kind of scenario occurs for supernova explosions.

### 4.3. Supernova Explosions

The stellar core continues collapsing until its density reaches the nuclear density. At the nuclear density, the core may explode if the inner pressure force term is greater than the gravitational force in Equation (54). This condition may be written as

(67) $$\frac{2GM}{R_{\min}}\leq \frac{32\pi \kappa \prime }{15MR_{\min}^{3\gamma -3}}$$

At the upper bound value of 2*GM*/*R*_min_, the potential energy becomes

(68) $$W(R_{\min})=\frac{\left(4-3\gamma \right)}{3(\gamma -1)}\frac{32\pi \kappa^{\prime }}{15M}\frac{1}{R_{\min}^{3\gamma -3}}=\frac{\left(4-3\gamma \right)}{3(\gamma -1)}\frac{2GM}{R_{\min}}$$

The potential energy given above is the total energy deposition during the core-collapse [6].

The equation of motion of the star corresponding to this potential energy is given by

(69) $$\frac{d^{2}}{dt^{2}}R(t)=(4-3\gamma )\frac{32\pi \kappa \prime }{15M}\frac{1}{R^{3\gamma -2}}$$

The right side of Equation (69) is always positive if *γ* is less than 4/3, in which case Equation (69) is an equation of motion for an expanding star. Consequently, the potential given in Equation (68) may be considered as the energy per unit mass that is needed for a protoneutron star to explode.

The solution of Equation (69) can be obtained using the aforementioned energy method. The solution for the case of *γ* = 7/6, which provides an explicit form of analytical solution for an expanding star, is given by [41]

(70) $$R=R_{\min}\cosh^{4}\theta$$

(71) $$t=\frac{R_{\min}}{\sqrt{2V(R_{\min})}}(\frac{\sinh4\theta }{8}+\sinh2\theta +\frac{3\theta }{2})$$

The explosion velocity of the protoneutron star can be obtained from Equations (70) and (71). That is,

(72) $$\frac{dR}{dt}=\sqrt{2V(R_{\min})}\tanh\theta =\sqrt{\frac{\left(4-3\gamma \right)}{3(\gamma -1)}\frac{2GM}{R_{\min}}}\tanh\theta$$

The explosion velocity whose asymptotic limit is $\sqrt{2GM/R_{\min}}$ generates a strong outgoing shock.

### 4.4. Heat Transport Equation for the Core-Collapse Supernova Explosions

During the collapsing phase of the star with *γ* < 4/3, the right side of Equation (65) becomes negative so that the heat flows radially outward. Thus, the heat flow from the whole volume of the star at a given time, which may be transported by radiation, is given by

(73) $$-\int_{0}^{R}(\nabla \cdot \vec{q})4\pi r^{2}dr=0.048\frac{\left(4-3\gamma \right)}{\left(\gamma -1\right)}\frac{GM^{2}}{R}\frac{\dot{R}}{R}$$

The above equation reveals that the heat flow rate is linearly dependent on the collapsing rate of the star when the core-mass and the specific heat ratio are given. The heat may radiate away by the neutrino flux, which can leave the star without interaction during the collapse of the supernova. The heat gain during the expansion phase may occur due to the absorption of neutrinos [42]. Solving the heat transport equation for the supernova given in Equation (42) is very hard task [35,36,37,43,44,45] because one can hardly know the processes inside the supernova during the core-collapsing explosions.

However, we can calculate the time rate change of heat flow from the core-collapsing supernova through Equation (73). The entropy generation rate when heat radiates away by neutrino flux can be written as

(74) S˙gen=1TavgdQdt−1T∞dQdt

where *T_avg_* is the instantaneous average temperature of the star. The entropy generation given in Equation (74) is positive because 1/*T*_∞_ is much greater than 1/*T_avg_*. On the other hand, when the star contracts, the entropy generation rate is given by

(75) S˙gen=1TavgdQdt

The entropy generation is again positive because the heat generates inside the supernova by the absorption of neutrinos. No heat flows into the star from outer space in this case.

**Figure 9:**
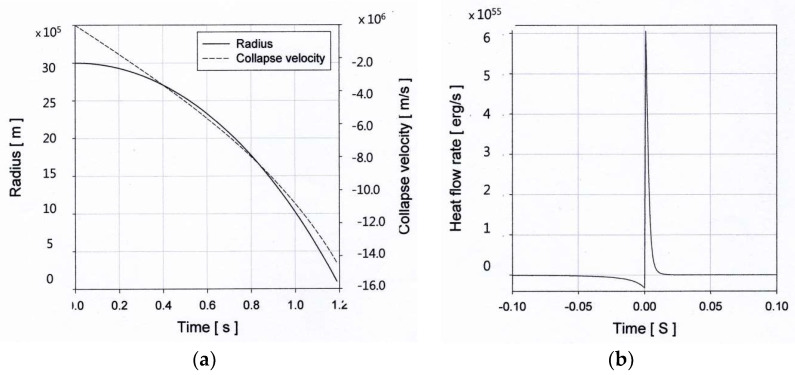
(**a**) The time-dependent radius and collapse velocity of an iron core; (**b**) the heat flow rate during the collapsing (**left**) and explosion (**right**) phase.

Figure 9a shows the time-dependent radius and the collapse velocity of a stellar iron-core having a mass of 1.5 M⊙. In this calculation, the initial radius and the specific heat ratio for the iron core were taken as 3000 km [42] and 1.2, respectively, and the density and temperature of the medium outside the core were taken as 10^6^ kg/m^3^ and 10^6^ K, respectively. The central density of 10^10^ kg/m^3^ and the central temperature of 6 × 10^10^ K (~509 keV), at which Fe-He transition can occur, were used in the calculation. The initial pressure estimated by the Wheeler equation of state for the progenitor star is about 10^23^ Pa [6]. However, an initial pressure of 1.2 × 10^24^ Pa for the star was used to satisfy the inequality given in Equation (58) in this study.

The collapse time and the heat flow are dependent on the specific heat ratios of the iron core. The shorter collapse time and larger heat flow from the star are obtained for a smaller value of the specific heat ratio. The calculated total of the heat flow from the iron core is as much as 5.9 × 10^51^ ergs during the collapse period of 1.2 s. At the final stage of the collapse, the calculated heat flow rate from the star is about 3.6 × 10^52^ ergs/s. This value is higher than the result obtained by Libendoerfer et al. [44] by one order of magnitude. The rate of the heat flow from the star is almost equal to the neutrino energy issued from the collapse, which is about 10^51^ ergs [45]. As can be seen in Figure 9b, the calculated energy input rate to the star at the earlier post-bounce is approximately 6.1 × 10^53^ ergs/s. This energy corresponds to the energy of the electron-type neutrinos emerging during the expansion phase. This calculation result is higher than other numerical calculations by an order of magnitude [36]. The calculated total of the energy input during expansion is about 10^52^ ergs. The heat flow into the expanding protoneutron star may occur through the absorption of neutrinos [42]. Without knowledge of the heat flow mechanism due to neutrino transport, the hydrodynamic equations, including the energy equation, predict the magnitude of the heat flow rate during the evolution of stars remarkably well.

## 5. Conclusions

The entropy generation due to heat transfer for evolving spherical objects such as mini and microbubbles and supernovae is discussed. The entropy generation due to heat transfer across the system boundary affects the delicate evolution of a bubble formed from a fully evaporated droplet at its superheat limit and sonoluminescing microbubbles under ultrasound. This is a case in which the heat transfer through the system boundary determines the evolution of the system. On the other hand, the heat transfer rate from supernovae is determined by their gravitational energy and evolving speed, which is the case in which the evolution of the system determines the heat transfer rate value through the system boundary [41]. All discussions in this article are based on the explicit solutions of the hydrodynamic equations such as mass, momentum and energy equations, and Newtonian gravitational equations for the homologically evolving spherical objects. The nonlinear behavior of sonoluminescing gas bubbles in sulfuric acid solutions and the heat transfer mechanism during the core-collapse supernova explosion cannot be obtained from the conventional approach such as the polytropic approximation for the bubble behavior and the assumption of an adiabatic process for the evolution of a star. The entropy generation rate is always positive for the evolving bubbles and supernova, which experience heat transfer.

## Nomenclature

*C_B_*	sound speed at the bubble wall
*C_p_*	heat capacity of star at constant pressure
*C_p,b_*	heat capacity of gas at constant pressure
*C_v_*	heat capacity of stat at constant volume
*C_v,b_*	heat capacity of gas at constant volume
*d_m_*	average distance between molecules
*d_w_*	van der Waals’ diameter of liquid molecule
*E*	internal energy
*f_d_*	driving frequency of ultrasound
*G*	gravitational constant
*k_g_*	conductivity of gas
*k_1_*	conductivity of liquid
*m_b_*	mass of bubble
*M*	mass of star
*N*	number density
*n*	polytropic index
*P*	pressure inside the star
*P_A_*	driving amplitude of ultrasound
*P_b_*	pressure inside the bubble
*P_bo_*	pressure at the bubble center
*P_s_*	driving ultrasound pressure
*P_o_*	pressure at the center of star
*P*_∞_	ambient pressure
*Q*	heat flow
*q_r_*	heat flux inside the bubble or star
*r_d_*	distance from the bubble center
*R*	radius of star
*R_o_*	equilibrium radius of bubble
*R_b_*	radius of bubble
*R_g_*	gas constant
*R*_min_	minimum radius of star
*R*_max_	maximum radius of star
*S*	entropy
*S_g_*	entropy generation
*t*	time
*T*	temperature of star
*T_avg_*	average temperature of star
*T_b_*	temperature of gas inside the bubble
*T_bl_*	temperature at the bubble-liquid interface
*T_bo_*	temperature at the bubble center
*T_o_*	temperature at the center of star
*T*_∞_	ambient temperature
*U_b_*	velocity at the bubble wall
*u*	velocity inside the star
*u_g_*	gas velocity inside the bubble
*V*	volume of bubble
*Vo*	initial volume of fire-ball
*V_m_*	effective volume of liquid molecule
*W*	potential for evolution of star
*Z*	coordination number

## Greek Letters

*α*	thermal diffusivity of liquid
*γ*	specific heat ratio
*δ*	thermal boundary layer thickness
*ε*	energy of fire-ball or star
*ε_m_*	energy needed to separate of pair of molecules
*ε_o_*	potential parameter of London dispersion force
*ρ*	density of star
*ρ_c_*	critical density of liquid
*ρ_g_*	gas density inside the bubble
*ρ_m_*	density of liquid
*ρ_o_*	gas density at the center of bubble or star
*ρ*_∞_	density of liquid medium
*ϕ*	gravitational potential
*ω*	angular frequency of ultrasound
*μ*	dynamic viscosity of liquid
*σ*	interfacial tension of liquid

## Subscripts

*b*	bubble
*o*	center
∞	ambient liquid medium

## References

[1] Bejan A. (1988). Advanced Engineering Thermodynamics.

[2] Moody F.J., Bejan A., Reid R.C. (1984). Second law thinking–example application in reactor and containment technology. Second Aspects of Thermal Design.

[3] Hopkins S.D., Putterman S.J., Kappus B.A., Suslick K.S., Camara C.G. (2005). Dynamics of a sonoluminescing bubble in sulfuric acid. Phys. Rev. Lett..

[4] Prosperetti A., Crum L.A., Commander K.W. (1988). Nonlinear bubble dynamics. J. Acoust. Soc. Am..

[5] Jun J.-H., Kwak H. (2000). Gravitational collapse of Newtonian stars. Int. J. Mod. Phys. D.

[6] Colgate S.A., White R.H. (1966). The hydrodynamics behavior of supernova explosions. Astrophys. J..

[7] Weinberg S. (1972). Gravitation and Cosmology.

[8] Oh J., Yoo Y., Seung S., Kwak H. (2018). Laser-induced bubble formation on a micro gold particle levitated in water under ultrasonic field. Exp. Therm. Fluid Sci..

[9] Blander M., Katz J.L. (1975). Bubble nucleation in liquids. AIChE. J..

[10] Shepherd J.E., Sturtevant B. (1982). Rapid evaporation at the superheat limit. J. Fluid Mech..

[11] Kwak H., Lee S. (1991). Homogeneous bubble nucleation predicted by a molecular interaction model. J. Heat Trans..

[12] Kwak H., Panton R.L. (1985). Tensile strength of simple liquids predicted by a model of molecular interactions. J. Phys. D Appl. Phys..

[13] Kwak H., Oh S., Park C. (1995). Bubble dynamics on the evolving bubble formed from the droplet at the superheat limit. Int. J. Heat Mass Trans..

[14] Theofanous T., Bias L., Isbin H.S. (1969). A theoretical study on bubble growth in constant and time-dependent pressure fields. Chem. Eng. Sci..

[15] Moody F.J. (1991). Non-intuitive bubble effects in reactor and containment technology. Fluids Engineering, Korea-U.S. Progress.

[16] Vincenti W.G., Kruger C.H. (1965). Infroduction to Physical Gas Dynamics.

[17] Rayleigh L. (1917). On the pressure developed in a liquid during the collapse of a spherical cavity. Phil. Mag..

[18] Gaitan D.F. (1990). An Experimental Investigation of Acoustic Cavitation in Gaseous Liquids. Ph.D. Thesis.

[19] Kwak H., Yang H. (1995). An aspect of sonoluminescence from hydrodynamic theory. J. Phys. Soc. Jpn..

[20] Lin H., Storey B.D., Szeri A.J. (2002). Inertially driven inhomogeneities in violently collapsing bubbles: The validity of the Rayleigh-Plesset equation. J. Fluid Mech..

[21] Kwak H., Na J.H. (1996). Hydrodynamic solutions for a sonoluminescing gas bubble. Phys. Rev. Lett..

[22] Kestin J., Knierim K., Masson E.A., Najafi B., Ro S.T., Waldman M. (1984). Equilibrium and transport properties of the noble gases and their mixtures at low density. J. Phys. Chem. Ref. Data.

[23] Byun K.-T., Kim K.Y., Kwak H. (2005). Sonoluminescence characteristics from micron and submicron bubbles. J. Korean Phys. Soc..

[24] Kim K.Y., Byun K.-T., Kwak H. (2006). Characteristics of sonoluminescing bubbles in aqueous solutions of sulfuric acid. J. Phys. Soc. Jpn..

[25] Delgadino G.A., Bonetto F.J. (1997). Velocity interferometry technique used to measure the expansion and compression phases of a sonoluminecent bubble. Phys. Rev. E.

[26] Keller J.B., Miksis M. (1980). Bubble oscillations of large amplitude. J. Acoust. Soc. Am..

[27] Kwak H., Na J.H. (1997). Physical processes for single bubble sonoluminescence. J. Phys. Soc. Jpn..

[28] Flannigan D.J., Hopkins S.D., Camara C.G., Putterman S.J., Suslick K.S. (2006). Measurement of pressure and density inside a single sonoluminescing bubble. Phys. Rev. Lett..

[29] Jeon J., Lim C., Kwak H. (2008). Measurement of pulse width of sonoluminescing gas bubble in sulfuric acid solution. J. Phys. Soc. Jpn..

[30] Gompf B., Gunther R., Nick G., Pecha R., Eisenmenger W. (1997). Resolving sonoluminescence pulse width with time-correlated single photon counting. Phys. Rev. Lett..

[31] Putterman S., Evans P.G., Vasquez G., Weninger K. (2001). Cavitation science: Is there a simple theory of sonoluminescence?. Nature.

[32] Hilgenfeldt S., Grossmann S., Lohse D. (2001). Hilgenfeldt et al.’s reply. Nature.

[33] Kim K.Y., Kwak H. (2007). Predictions of bubble behavior in sulfuric acid solutions by a set of solutions of Navier-Stokes equations. Chem. Eng. Sci..

[34] Jeans J. (1902). The stability of a spherical nebula. J. Phil. Trans. Roy. Soc..

[35] Rampp M., Janka H.-T. (2002). Radiation hydrodynamics with neutrinos: Variable Eddington factor method for core-collapse supernova simulations. Astron. Asrophys..

[36] Livne E., Burrows A., Walder R., Lichtenstadt I., Thompson T.A. (2004). Two-dimensional, time-dependent, multigroup, multiangle radiation hydrodynamics test simulation in the core-collapse supernova context. Astrophys. J..

[37] Buras R., Rampp M., Janka H.-Th., Kifonidis K. (2006). Two-dimensional hydrodynamics core-collapse supernova simulations with spectral neutrino transport. I. Numerical method and results for a 15 M_⊙_ star. Astron. Asrophys..

[38] Murphy J.W., Burrows A. (2008). Criteria for core-collapse supernova explosions by the neutrino mechanism. Astrophys. J..

[39] Kwak H., Jun J.-H. (2003). Hydrodynamics and thermodynamics for Newtonian stars. Geophys. Astrophys. Fluid Dyn..

[40] Oppenheimer J.R., Snyder H. (1939). On continued gravitational contraction. Phys. Rev..

[41] Kwak H. (2015). Core-collapse supernova explosions: An analytical one-dimensional analysis. Far East J. Appl. Math..

[42] Burrows A. (2000). Supernova explosions in the Universe. Nature.

[43] Burrows A., Hays J., Fryxell B.A. (1995). On the nature of core-collapse supernova explosions. Astrophys. J..

[44] Liebendoerfer M., Rampp M., Janka H.-T., Mezzacappa A. (2005). Supernova simulations with Boltzmann neutrino transport: A comparison of methods. Astrophys. J..

[45] Nordhaus J., Burrows A., Almgren A., Bell J. (2010). Dimension as a key to the neutrino mechanism of core-collapse supernova explosions. Astrophys. J..

